# Association of apolipoprotein E polymorphism with maximal oxygen uptake after exercise training: a study of Chinese young adult

**DOI:** 10.1186/1476-511X-13-40

**Published:** 2014-02-27

**Authors:** Bo Yu, Wenhua Chen, Renwei Wang, Qi Qi, Kunpeng Li, Wen Zhang, Huiru Wang

**Affiliations:** 1 School of Kinesiology, Shanghai University of Sport, Shanghai 200438, China; 2 Department of Rehabilitation Medicine, Shanghai First People’s Hospital, Shanghai Jiao Tong University, Shanghai 200080, China; 3 Department of Physical Education, Shanghai Jiao Tong University, Shanghai 200240, China

**Keywords:** Apolipoprotein E, Gene polymorphism, Maximal oxygen uptake, Exercise training

## Abstract

**Background:**

Although a few studies have been conducted, it is still unclear whether the apolipoprotein E (*APOE*) polymorphism is associated with maximal oxygen uptake (VO_2max_) after exercise training. The objective of this study was to examine if the *APOE* gene polymorphisms affect VO_2max_ after exercise training in Chinese young adult.

**Methods:**

A total of 360 Chinese young adult (180 male and 180 female) were recruited into this gender-specific cohorts. Anthropometrics, serum lipids, and VO_2max_ were measured pre and post 6 months of supervised exercise training. Polymerase chain reaction restriction fragment length polymorphism (PCR-RFLP) assay was applied to assess the *APOE* gene polymorphisms.

**Results:**

VO_2max_ after exercise training increased significantly higher in carriers of E2/E3 in male [odds ratio (OR) =0.68, 95% confidence interval (CI) = 0.04, 1.32; *P* = 0.04] and female (OR =0.62, 95% CI = 0.05, 1.18; *P* = 0.03). VO_2max_ after exercise training increased significantly higher in carriers of E3/E4 in male (OR =0.60, 95% CI = 0.09, 1.11; *P* = 0.02) and female (OR =0.62, 95% CI = 0.09, 1.15; *P* = 0.02). No significant differences were found in carriers of E2/E2, E2/E4, E3/E3, E4/E4 in either male nor female.

**Conclusion:**

Our study found that *APOE* gene polymorphism was associated with VO_2max_ levels after exercise training in Chinese young adult. In the future, further experiments will be necessary to confirm this finding and to find the possible mechanism.

## Introduction

Physical performance is a complex phenotype influenced by both environmental and genetic factors [1]. Great attention is paid to searching genes underlying athletic performance and variants predisposing to certain sport disciplines [1-4]. Changes in serum lipids with exercise training are often small and individually variable, limiting the role of exercise in treating lipid abnormalities [5]. Maximal oxygen uptake (VO_2max_) is traditionally recognized as the gold standard laboratory measure of cardio respiratory fitness, with increasing levels accompanying endurance training and high levels being a pre-requisite for success in endurance events [6].

Apolipoprotein E (apoE), a 299-amino acid, arginine-rich glycoprotein, is an integral surface component of chylomicrons, very-low-density lipoproteins (VLDL), and some subclasses of high-density lipoproteins (HDL). The *APOE* gene, encoded on chromosome 19, exists in three isoforms: E2, E3, and E4, giving six possible genotypes (E2/E2, E2/E3, E2/E4, E3/E3, E3/E4 and E4/E4) [7-9]. *APOE* gene polymorphisms seem to have some impact among patients with cardiovascular disease [10-12].

Although a few studies have been conducted, it is still unclear whether the *APOE* polymorphism is associated with VO_2max_ after exercise training [13,14]. The objective of this study was to examine if the *APOE* gene polymorphisms affect VO_2max_ after exercise training in Chinese young adult.

## Materials and methods

### Study population

A total of 360 Chinese young adult (180 male and 180 female) were recruited into this gender-specific cohorts during the years 2012 to 2013 from Shanghai University of Sport, China. Anthropometrics, serum lipids, and VO_2max_ were measured pre and post 6 months of supervised exercise training. Eligibility criteria included requirements for participants to be healthy, physically inactive, 18–40 years old, to pass a physician-administered physical examination, and to have no significant electrocardiographic abnormalities during a cycle ergometer maximal exercise test. Subjects were considered physically inactive if they participated in vigorous activity fewer than 4 times per month for the prior 6 months. Exclusion criteria included diabetes mellitus, hypertension, hyperlipidemia, or a body mass index (BMI) exceeding 30 kg/m^2^. Subjects underwent a progressive, supervised exercise training program. Subjects exercised between 60% and 85% of their VO_2max_ based on their predetermined maximal heart rate. VO_2max_ was defined as the average of the 2 highest consecutive 30-second values at peak exercise. Treadmill exercise was the primary mode of training. Subjects were requested to maintain their usual dietary composition throughout the study. The Ethical Committee of the Shanghai University of Sport approved the study protocols, and all participants gave written informed consent according to the Declaration of Helsinki. Each subject received an incremental honorarium for successful completion of the study.

### DNA extraction and genotyping

Venous blood samples were collected in EDTA-containing tubes from each participant. DNA was extracted from peripheral blood leukocytes using the phenol-chloroform method. Polymerase chain reaction restriction fragment length polymorphism (PCR-RFLP) assay was applied to assess the *APOE* gene polymorphisms. Based on the GenBank reference sequence, the PCR primers were as follows: sense, ACAGAATTCGCCCCGGCCTGGTACAC; antisense, TAAGCTTGGCACGGCTGTCCAAGGA. The amplified PCR products were then digested with 2U of *Hha* I (New England BioLabs, Missisauga, ON, Canada) at 37°C for 3 hours. The resulting DNA fragments were electrophoresed on 3.5% agarose gel and visualized under UV light after ethidum staining.

### Statistical analysis

All analyses were performed with Statistical Analyses System (SAS) package (version 8.01; SAS Institute, Cary, NC). Univariate analyses of variance were used to compare *APOE* groups for differences at baseline and differences in their responses to exercise training. The *P* value of statistical significance was adjusted by Fisher’s exact test where appropriate. A *P*-value was considered significant at a level of < 0.05. We calculated crude and adjusted odds ratios (OR) and 95% confidence intervals (CI) for the association between the *APOE* genotypes and VO_2max_ after exercise training. A chi-square test was used to confirm that the *APOE* genotype frequencies were in Hardy-Weinberg equilibrium.

## Results

The demographical and physiological characteristics of the participants were showed in Table 1. The mean age was 32.8 (±11.9) years for the male and 31.0 (±12.0) years for the female. The body weight was 75.1 (±15.8) kg for the male and 69.7 (±13.1) kg for the female. The BMI was 25.4 (±5.6) kg/m^2^ for the male and 26.0 (±6.2) kg/m^2^ for the female. The waist-to-hip ratio (WHR) was 0.81 (±0.08) for the male and 0.75 (±0.06) for the female. The VO_2max_ was 2.62 (±0.51) L/min or 34.9 (±6.8) mL/kg/min for the male and 1.85 (±0.36) L/min or 26.5 (±5.2) mL/kg/min for the female. The total cholesterol (TC) was 171.4 (±6.4) mg/dL for the male and 163.5 (±5.7) mg/dL for the female. The low-density lipoprotein (LDL-C) was 116.1 (±4.8) mg/dL for the male and 112.2 (±4.1) mg/dL for the female. The HDL-C was 45.1 (±1.6) mg/dL for the male and 49.2 (±1.8) mg/dL for the female. The VLDL-C was 10.2 (±0.7) mg/dL for the male and 12.1 (±0.8) mg/dL for the female. The triglycerides (TG) was 106.5 (±13.2) mg/dL for the male and 89.7 (±10.2) mg/dL for the female (Table 1).

**Table 1:** The demographical and physiological characteristics of the participants

	**Male**	**Female**
Total No.	180	180
Age (year)	32.8 ± 11.9	31.0 ± 12.0
Body weight (kg)	75.1 ± 15.8	69.7 ± 13.1
BMI (kg/m^2^)	25.4 ± 5.6	26.0 ± 6.2
WHR	0.81 ± 0.08	0.75 ± 0.06
Vo_2_max (L/min)	2.62 ± 0.51	1.85 ± 0.36
Vo_2_max (mL/kg/min)	34.9 ± 6.8	26.5 ± 5.2
TC (mg/dL)	171.4 ± 6.4	163.5 ± 5.7
LDL-C (mg/dL)	116.1 ± 4.8	112.2 ± 4.1
HDL-C (mg/dL)	45.1 ± 1.6	49.2 ± 1.8
HDL_2_-C (mg/dL)	14.0 ± 1.0	16.1 ± 1.1
HDL_3_-C (mg/dL)	31.1 ± 1.8	33.1 ± 1.9
VLDL-C (mg/dL)	10.2 ± 0.7	12.1 ± 0.8
TG (mg/dL)	106.5 ± 13.2	89.7 ± 10.2
apo A1 (mg/dL)	118.4 ± 3.5	125.3 ± 3.9
apo B (mg/dL)	85.9 ± 5.8	75.2 ± 4.9

BMI, body mass index; WHR, waist-to-hip ratio; Vo_2_max, maximal oxygen uptake; TC, total cholesterol; LDL, low-density lipoprotein; HDL, high-density lipoprotein; VLDL, very-low-density lipoprotein; TG, triglycerides.

VO_2max_ after exercise training increased significantly higher in carriers of E2/E3 in male (OR =0.68, 95% CI = 0.04, 1.32; *P* = 0.04) and female (OR =0.62, 95% CI = 0.05, 1.18; *P* = 0.03) (Tables 2 and 3). VO_2max_ after exercise training increased significantly higher in carriers of E3/E4 in male (OR =0.60, 95% CI = 0.09, 1.11; *P* = 0.02) and female (OR =0.62, 95% CI = 0.09, 1.15; *P* = 0.02) (Tables 2 and 3). No significant differences were found in carriers of E2/E2, E2/E4, E3/E3, E4/E4 in either male nor female.

**Table 2:** **Frequencies of *APOE *** **gene polymorphisms and maximal oxygen uptake (L/min) pre and post exercise training (mean ± SD) in male**

**Genotype**	**Male**	**Pre**	**Post**	**OR (95% CI)**	***P***
E2/E2	7	2.65(0.57)	2.79(0.51)	0.26(-0.79,1.31)	0.63
E2/E3	20	2.61(0.50)	2.97(0.56)	0.68(0.04,1.32)	0.04
E2/E4	6	2.66(0.55)	2.83(0.51)	0.32(-0.82,1.46)	0.58
E3/E3	110	2.61(0.50)	2.70(0.52)	0.18(-0.09,0.44)	0.19
E3/E4	31	2.63(0.52)	2.95(0.55)	0.60(0.09,1.11)	0.02
E4/E4	6	2.67(0.54)	2.80(0.51)	0.25(-0.89,1.38)	0.67

OR, odds ratio; CI, confidence interval.

**Table 3:** **Frequencies of *APOE *** **gene polymorphisms and maximal oxygen uptake (L/min) pre and post exercise training (mean ± SD) in female**

**Genotype**	**Female**	**Pre**	**Post**	**OR (95% CI)**	***P***
E2/E2	9	1.87(0.36)	1.95(0.39)	0.21(-0.71,1.14)	0.65
E2/E3	25	1.86(0.38)	2.10(0.40)	0.62(0.05,1.18)	0.03
E2/E4	8	1.88(0.37)	1.93(0.36)	0.14(-0.84,1.12)	0.78
E3/E3	102	1.85(0.36)	1.90(0.38)	0.14(-0.14,0.41)	0.34
E3/E4	29	1.84(0.35)	2.07(0.39)	0.62(0.09,1.15)	0.02
E4/E4	7	1.86(0.38)	1.97(0.37)	0.29(-0.76,1.35)	0.59

OR, odds ratio; CI, confidence interval.

## Discussion

A lot of studies have been conducted to examine the association of genetic polymorphism and athletic performance. A recent study found that genetic variants of uncoupling proteins-2 and -3 were associated with VO_2max_ in different sports [1]. A study in 323 Russian athletes and 467 nonathletic controls found that monocarboxylate transporter 1 gene A1470T polymorphism was associated with VO_2max_[15]. A cohort of 67 Chinese men in Singapore suggested that the angiotensin-converting enzyme (*ACE*) DD genotype in young adult Chinese males was associated with higher levels of VO_2max_[16]. The *ACE* I/D polymorphism altered the response of muscle energy supply lines to exercise [17]. *AKT1* G205T genotype influenced obesity-related metabolic phenotypes and their responses to aerobic exercise training in older Caucasians [18]. The Genathlete cohort found preliminary evidence that the hypoxia-inducible factor-1alpha Pro582Ser polymorphism may be associated with elite endurance athletes in Caucasian men [19]. A study in 1,423 Russian athletes and 1,132 controls suggested that the likelihood of becoming an elite endurance athlete depended on the carriage of a high number of endurance-related alleles [20]. The A2962G polymorphism of the peroxisome proliferator-activated receptor gamma coactivator 1 alpha (*PPARGC1A*) gene was associated with VO_2max_ at baseline, as carriers of the G allele had higher levels of VO2_max_ than the AA group endurance capacity in Chinese men [21]. The HERITAGE Family Study found that peroxisome proliferator-activated receptor-delta (*PPARdelta*) polymorphisms were associated with physical performance and plasma lipids [22].

The *APOE* gene polymorphisms were associated with diseases of the respiratory system and cardiovascular disease. Small lung volumes were prospectively associated with an increased risk for poor cognitive function and dementia in non-carriers of the *APOE* E4 [23]. Studies in transgenic mice showed that alpha-tocopherol transport in the lung was affected by the *APOE* genotype [24]. *APOE* E4 and cardiorespitatory fitness could interact to influence child adiposity in 8-year-old children from the Tasmanian Infant Health Survey [25]. Although meta-analyses suggest that *APOE* E4 carriers may have a 40–50% increased coronary artery disease risk, the associations reported in individual studies are highly heterogeneous [26]. In the Tunisian population the *APOE* E4 appears to be only indirectly involved in the severity of cardiovascular disease [27]. Although the prevalence of the *APOE* E4 allele is generally low, there are areas with higher prevalence of the *APOE* E4 allele and a higher incidence of adult ischemic heart disease mortality in Spain [28]. An autopsy study suggested that the risk of developing and dying from cardiovascular disease, including coronary heart disease and cerebrovascular disease, was influenced by the *APOE* polymorphism [29]. The *APOE* E2 genotype might contribute to increased risk of cardiovascular complications in subjects with acromegaly [30]. The *APOE* genotype predicted cardiovascular endpoints in dialysis patients with type 2 diabetes mellitus [31]. A meta-analysis of 45 studies including 13,940 cases and 16,364 controls found that *APOE* gene polymorphisms were associated with essential hypertension [32].

How did the *APOE* gene polymorphisms affect VO_2max_ after exercise training? The exact mechanism behind it is still unclear. There is animal evidence that Apo E can affect exercise performance [33]. Controversy exists as to relationship of *APOE* polymorphism to the blood lipid response to exercise [34,35]. It was unlikely to explain the present results. Apo E mRNA is expressed in skeletal muscle and appears to be most abundant at neuromuscular junctions [36]. Therefore the effect of *APOE* genotype on exercise capacity may have been mediated by more direct effects on other tissues such as skeletal muscle [14].

There are some limitations to the present study that should be noted. First of all, the present study lacked a control group, since this is a self-control study. Second, the sample size of this study is relatively small, which may not have enough statistical power to explore the real association. Third, we cannot exclude the possibility that some other genetic factor associated with *APOE* variants is responsible for the differences in the VO_2max_ response. Finally, these results should be interpreted with caution because the population was only from China, which reduces the possibility of confounding from ethnicity, so it does not permit extrapolation of the results to other ethnic groups.

## Conclusion

In conclusion, our study found that APOE gene polymorphism was associated with VO2max levels after exercise training in Chinese young adult. In the future, further experiments will be necessary to confirm this finding and to find the possible mechanism.

## Competing interest

The authors declare that they have no competing interests.

## Authors’ contributions

BY and WC carried out the molecular genetic studies and drafted the manuscript. RW and QQ carried out the genotyping. KL and WZ participated in the design of the study and performed the statistical analysis. BY, WC and HW conceived of the study, and participated in its design and coordination and helped to draft the manuscript. All authors read and approved the final manuscript.

## Authors’ information

Bo Yu and Wenhua Chen are joint first authors.
